# Gut Microbiota Dynamics and Uremic Toxins

**DOI:** 10.3390/toxins14020146

**Published:** 2022-02-17

**Authors:** Eikan Mishima, Takaaki Abe

**Affiliations:** 1 Division of Nephrology, Endocrinology, and Vascular Medicine, Tohoku University Graduate School of Medicine, Sendai 980-8574, Japan; taka@med.tohokua.ac.jp; 2 Division of Medical Science, Tohoku University Graduate School of Biomedical Engineering, Sendai 980-8574, Japan; 3 Department of Clinical Biology and Hormonal Regulation, Tohoku University Graduate School of Medicine, Sendai 980-8574, Japan

Recent evidence has highlighted the importance of the gut microbiota in the pathophysiology of kidney diseases [1,2,3]. The gut microbiota is involved in the production of many uremic toxins, such as indoxyl sulfate, *p*-cresyl sulfate, and trimethylamine *N*-oxide (TMAO), which are retained in chronic kidney disease (CKD) [4]. Retention of these uremic solutes results in a variety of symptoms such as cardiovascular dysfunction, pruritus, fatigue, renal anemia, mineral bone disorder, and neurological impairment, all of which are symptomatic in CKD patients [5,6,7]. The microbiota also produce beneficial metabolites for the host, such as short-chain fatty acids [8]. Altered composition of the gut microbiota affects the plasma levels of these uremic solutes in CKD. Thus, modulating intestinal microbiota by molecules such as antibiotics and pre- and probiotics, nonlethal inhibition of microbial-specific enzymes, and pharmacological approaches targeting the intestine can be an interesting approach to control uremic symptoms and the resulting disease [2,3,9,10,11]. The relationship between the microbiota and uremic toxins in kidney and cardiovascular diseases is currently under investigation. This Special Issue, entitled “Gut Microbiota Dynamics and Uremic Toxins”, focuses on the connection between microbiota and uremic toxins.

Graboski and Redinbo [12] have reviewed the derivation and pathological mechanisms of gut-derived, protein-bound uremic toxins and outlined the emerging association between kidney disease and gut dysbiosis. The considered aspects were altered bacterial taxa, regulation of microbial uremic toxin-producing genes, and their downstream physiological and neurological consequences.

Taguchi et al. [13] and Rysz et al. [14] have summarized the underlying mechanism of gut microbial dysbiosis promoting kidney injury. These studies highlighted the wide-ranging interventions to counter dysbiosis in CKD patients from the perspective of uremic toxins, including TMAO and advanced glycation end products. TMAO is a potent pro-atherosclerotic and pro-thrombotic uremic toxin, generated by microbiota metabolism. Advanced glycation end products cause gut dysbiosis by disrupting the intestinal barrier.

El Amouri et al. [15] have found an inverse association between increased fiber consumption and serum levels of gut-derived protein-bound uremic toxins in pediatric patients with CKD, highlighting the potential benefits of fiber intake for the pediatric CKD population.

Barba et al. [16] explored whether fecal microbiota transplantation (FMT) could attenuate metabolic complications and uremic toxin accumulation in mice with CKD. Compared to control mice, the mice that received FMT showed increased gut microbiota diversity, decreased p-cresyl sulfate accumulation, and improved glucose tolerance.

Mishima et al. [17] examined the impact of microbiota on purine metabolism and its involvement in an adenine-induced CKD model using germ-free mice. The germ-free mice displayed higher levels of expression of purine-metabolizing enzymes, such as xanthine dehydrogenase, which converts adenine to the nephrotoxic byproduct 2,8-dihydroxyadenine (2,8-DHA), compared with the mice with microbiota. Enhanced host purine metabolism in germ-free mice promoted the conversion of the administered adenine into 2,8-DHA, resulting in exacerbated kidney damage in the CKD model. The study findings further suggest an important role of the microbiota in regulating host purine metabolism.

In addition to CKD, the microbiota and intestinal environment are involved in the pathophysiology of acute kidney injury (AKI) and cardiovascular diseases including hypertension [18]. In a review article, Kobayashi et al. [19] have discussed the pathogenesis of AKI, highlighting its relationship with gut microbiota. Some gut bacteria and their metabolites have been reported to exert protective effects against AKI. Yamashita et al. [20] have investigated the evidence from several clinical and experimental studies that indicated an association between gut-microbiota-derived toxins and cardiovascular diseases. The authors focused on the pro-inflammatory gut-microbiota-derived toxins, namely, lipopolysaccharides, derived from gram-negative bacteria, and TMAO.
